# Impact of Pycnogenol® Use in the Treatment of Patients With Lipedema: A Randomized Controlled Trial

**DOI:** 10.7759/cureus.96589

**Published:** 2025-11-11

**Authors:** Brenno Augusto S Mello Netto, José Marcelo Corassa, Fanilda S Barros, Vanessa Zani, Milena S Seabra de Mello, Moriane B Lorenzoni, Nadia M Assis

**Affiliations:** 1 Vascular Surgery, Seabra Excelência Vascular, Vitória, BRA; 2 Vascular Surgery, Santa Paula Hospital, Vitória, BRA; 3 Vascular Surgery, Instituto Fanilda Barros, Vitória, BRA; 4 Nutrition, Seabra Excelência Vascular, Vitória, BRA; 5 Physiotherapy, Seabra Excelência Vascular, Vitória, BRA

**Keywords:** lipedema, polyphenol, pycnogenol®, quality of life (qol), randomized control trial

## Abstract

Aim

Lipedema is a chronic, progressive disease that predominantly affects women and is strongly influenced by estrogen. Its onset or worsening is associated with hormonal periods such as puberty, pregnancy, pregnancy induction, and menopause. It is characterized by abnormal, bilateral fat deposition in the buttocks and legs, which may be accompanied by edema, pain, and tenderness to touch. It is still frequently confused with more common conditions such as obesity and lymphedema. Pycnogenol® is a combination of anthocyanins and phenolic acids belonging to the large polyphenol family, with potent antioxidant activity and a proven effect in controlling chronic venous insufficiency. It has been used by several professionals for the symptomatic management of lipedema.

Objectives

This double-blind, randomized clinical trial sought to demonstrate the effectiveness of Pycnogenol® in the symptomatic control and body composition management of patients with lipedema.

Methods

This was a double-blind, randomized clinical trial with 60 days of follow-up involving one hundred patients. The study utilized a quality-of-life questionnaire (QuASiL), bioimpedance analysis, and clinical monitoring.

Results

Of the one hundred patients initially included, seven were lost to follow-up; however, monotonic multiple imputation was applied for data analysis. The two groups were similar in all aspects except for initial weight. The placebo group showed an increase in mean QuASiL scores after 30 and 60 days from the first assessment, representing a worsening of symptoms over time. In contrast, the intervention group demonstrated a progressive and significant reduction in scores, with means of 69.5 ± 28 at 30 days and 63.2 ± 27 at 60 days (p < 0.001). This group also showed a statistically significant reduction in weight, BMI, and body fat percentage.

Conclusions

Pycnogenol® appears to be a promising therapeutic option to support the clinical management of lipedema, a condition that exerts numerous negative physical and emotional impacts throughout the lives of affected patients.

## Introduction

Lipedema is a chronic and progressive condition characterized by the abnormal accumulation of fat, which can significantly impact the quality of life of women [1]. It manifests disproportionately and nodularly, mainly in the lower limbs, although it may also affect the lower abdomen, hips, buttocks, and upper limbs. The trunk, hands, and feet are usually spared. This peculiar distribution can cause physical and emotional challenges, affecting mobility and self-esteem [2].

There are still many gaps in knowledge regarding its true prevalence, risk factors, and pathophysiology. Evidence suggests that genetic and hormonal factors play a crucial role in the development of lipedema, with a higher incidence during periods of hormonal change such as puberty, pregnancy, pregnancy induction, and menopause. These triggers are often associated with estrogen predominance, which may influence fat distribution and fluid retention [3-5]. As a result, the predominance in females is striking, with an estimated prevalence of 11-39% in this group [6]. Studies have also investigated its association with lifestyle factors such as diet and physical activity [7].

The diagnosis of the disease remains challenging due to its complex pathophysiology and the absence of specific genetic markers. Evaluation is based predominantly on the patient’s clinical history and physical examination [8,9]. The overlap of signs and symptoms with other conditions, such as overweight, obesity, lymphedema, and chronic venous disease, further contributes to diagnostic complexity. These conditions often coexist, making clinical distinction difficult and leading to underdiagnosis, which consequently underestimates the prevalence of lipedema in medical practice [10].

Understanding the main signs and symptoms is therefore extremely relevant for the appropriate management of cases. The most frequently observed manifestations include pain (resulting from the inflammatory process and tissue fibrosis), hypersensitivity in affected areas, persistent edema, chronic fatigue, spontaneous bruising, and hypermobility due to increased joint flexibility [7,10,11].

The clinical classification of lipedema is based on the morphological assessment of the lower limbs. Recent diagnostic criteria include the accumulation of subcutaneous adipose tissue with a gynecoid pattern, resistance to weight loss, bilateral persistent edema in the legs (even with elevation), and the presence of palpable subcutaneous nodules [8]. The disease can be categorized according to the anatomical distribution of adipose tissue, classified into five types, and stratified into clinical stages ranging from I to IV, corresponding to the severity of morphological changes observed during inspection and palpation [9].

The literature highlights the importance of a multidisciplinary approach and surgical treatment [2]. However, there remains an urgent need to investigate less invasive options, as studies focusing on this aspect are scarce.

In this context, agents that positively influence blood and lymphatic microcirculation have emerged as effective options to alleviate symptoms such as pain and edema, which are very common in lipedema [7]. Pycnogenol® stands out as a relevant candidate for clinical practice, given its action on central pathophysiological mechanisms of the disease such as inflammation, oxidative stress, and vascular fragility. With proven efficacy in the treatment of vascular fragility in the lower limbs, prevention of complications caused by chronic venous disease and traveler’s syndrome, as well as in the treatment of hemorrhoidal disease, this standardized extract from the bark of Pinus pinaster is rich in polyphenols, procyanidins, phenolic acids, cinnamic acid, and its derivatives. Thus, it exerts anti-inflammatory, antioxidant, lipolytic, and venotonic effects, among others, giving it a potential pharmacological profile suitable for the treatment of this condition [9,10,12].

The search for effective pharmacological treatments for lipedema is both necessary and urgent. Therefore, this study aimed to investigate the efficacy of Pycnogenol® in improving quality of life and anthropometric parameters in women with lipedema. This validation is crucial to expanding therapeutic options and improving disease management.

## Materials and methods

Study design

This was a double-blind, randomized, placebo-controlled clinical trial consisting of two groups: intervention (Pycnogenol® 50 mg - marketed under the trade name Flebon®) and placebo.

Placebo composition

The placebo administered to patients had the same characteristics in terms of packaging, shape, color, texture, and odor as the control medication (Flebon). The chemical composition of the placebo was identical to that of Flebon, except for the absence of Pycnogenol. Because Pycnogenol imparts color to the tablet, a dye was used to maintain visual similarity between the placebo and control tablets.

Population

The number of patients required to conduct the study (97) was determined using a sample size calculation formula based on the use of a data collection instrument composed of categorical items. A population size of one million inhabitants was used as a parameter, with a 95% confidence level and a 10% margin of error. The final sample was non-probabilistic and comprised 100 women between 18 and 40 years of age, residents of Vitória, Espírito Santo, with a clinical diagnosis of lipedema, regardless of disease stage. The diagnosis was confirmed by two angiologists. Exclusion criteria included pregnancy, lactation, use of anabolic steroids or appetite suppressants, and patients already undergoing lipedema treatment with another professional or engaged in dietary interventions aimed at weight loss.

Sample size

$\frac{ \frac{z^{2} \cdot p (1 - p)}{e^{2}} }{ 1 + \left( \frac{z^{2} \cdot p (1 - p)}{e^{2} \cdot N} \right) }$

Data collection

**Figure 1:**
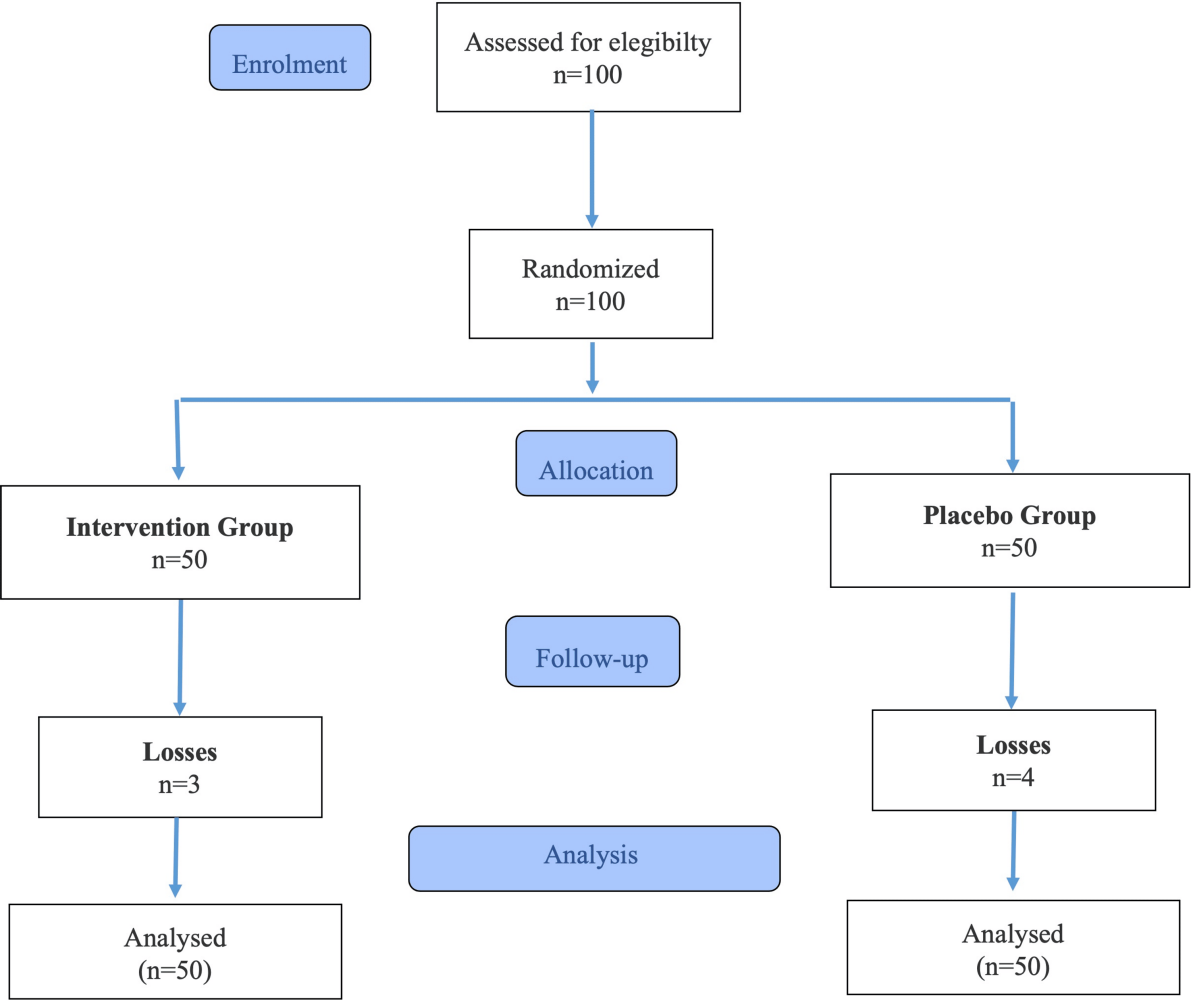
CONSORT flowchart (2025): recruitment, randomization, and data analysis. CONSORT: Consolidated Standards of Reporting Trials.

After verbal authorization and signing of the Informed Consent Form, each patient was randomly allocated to one of the two groups, as shown in Figure 1. Randomization was performed using a list of random numbers generated at sealedenvelope.com [13], where the number of treatments (intervention and placebo) and the number of patients (n = 50) in each block were entered (simple randomization method). To ensure proper study design and blinding, all volunteers received blank boxes labeled with the letters “A” or “B,” containing the lot number, manufacturing and expiration dates, and usage instructions. The boxes contained the same quantity of tablets identical in color, shape, size, and odor, making them indistinguishable and thereby ensuring blinding for both participants and researchers.

Each volunteer received enough tablets for use until the next visit (84 tablets in total), with instructions for administration every 8 hours. All medications were manufactured at the same facility and supplied by the FQM Farma Group. Additionally, support was provided by Horphag Research.

The research was conducted between April 15 and June 24, 2024, at a specialized clinic in Vitória (ES). Each patient attended three in-person visits with the researchers, the first for screening and inclusion, and the subsequent ones after 4 and 8 weeks. In addition, weekly telephone calls were made to ensure adherence and address any questions regarding medication use.

Evaluations were carried out by a multidisciplinary team consisting of physicians and a nutritionist. The initial evaluation included a structured questionnaire covering personal data, sociodemographic profile, lifestyle habits, health conditions, regular medication use, family history of lipedema and other chronic diseases, sleep quality, and the impact of lipedema on self-esteem and marital relationships. Additionally, participants answered the Lipedema Symptom Assessment Questionnaire (QuASiL) [14], a validated tool to measure symptom intensity. At follow-up visits, participants repeated the same assessments, except for the sociodemographic inventory.

Outcome evaluation

The primary outcome was the impact of Pycnogenol® on quality of life in women with lipedema, measured through the QuASiL questionnaire. This tool, adapted and validated for the Brazilian population by Amato AC et al. [14], consists of 15 self-reported questions scored from 0 to 10, covering symptoms such as pain, fatigue, burning sensation, swelling, and satisfaction with leg appearance. Scores range from 0 to 150, with higher values indicating a greater symptom impact on quality of life.

Body composition assessment

The secondary outcome was assessed through total and segmental body composition using tetrapolar bioimpedance (InBody120, InBody Co., Seoul, Korea). Body weight, total body fat percentage, and fat distribution in the upper and lower limbs were measured.

Statistical analysis

The Kolmogorov-Smirnov test was used to assess the normality of continuous variables. Descriptive statistics were expressed as mean ± standard deviation for symmetric continuous variables and as median and interquartile ranges for asymmetric continuous variables. Categorical variables were expressed as frequency and percentage.

Group comparisons at baseline were performed using the Kruskal-Wallis test, Fisher’s exact test, or univariate ANOVA, as appropriate. To assess treatment effects (within-group, between-group, and time-group interaction), repeated-measures ANOVA was applied. A significance level of 5% (p < 0.05) was adopted for all analyses. Statistical analyses were conducted using SPSS version 24.0.

Ethics

The study complied with Resolution 466/2012, respecting the principles of autonomy, non-maleficence, beneficence, justice, and equity. Confidentiality and privacy were maintained, preserving the identity of participants. The study was approved by the Research Ethics Committee (approval number 6.703.630; CAAE: 76906723.1.0000.5061). This trial was registered in the Brazilian Clinical Trials Registry under RBR-6bcnbpq.

## Results

A total of 100 women met the eligibility criteria and were randomized at baseline. Seven participants dropped out (three from the intervention group and four from the placebo group), leaving 93 participants who completed the study. To address these losses, monotone multiple imputation was applied for data analysis.

The median age was 35 years (IQR: 32-38), with no statistically significant difference between the groups. Specifically, in the placebo group, the median was 35 years (31-38), and in the intervention group, 34 years (32-38). As described in Table 1, sociodemographic characteristics were similar between the groups. Most women self-identified as white or mixed race, and only 6% of participants self-identified as black. The majority of participants were married and reported having had at least one pregnancy. All women evaluated denied habits such as smoking and illicit drug use, and 52% reported social consumption of alcoholic beverages.

**Table 1:** Sociodemographic characteristics, lifestyle habits, and lipedema symptoms at baseline. ¹ Fisher’s exact test applied.

Variable	Total	Placebo	Intervention (Pycnogenol®)	p-value
Skin Color				
White	46 (46%)	20 (40%)	26 (52%)	0.249^1^
Mixed	48 (48%)	28 (56%)	20 (40%)	
Black	6 (6%)	2 (4%)	4 (8%)	
Marital Status				
Married	65 (65%)	34 (68%)	31 (62%)	0.529
Unmarried	35 (35%)	16 (32%)	19 (38%)	
Pregnancies				
No	43 (43%)	18 (36%)	25 (50%)	0.157
Yes	57 (57%)	32 (64%)	25 (50%)	
Alcohol Consumption				
No	48 (48%)	23 (46%)	25 (50%)	0.841
Yes	52 (52%)	27 (54%)	25 (50%)	
Physical Activity				
No	27 (27%)	15 (30%)	12 (24%)	0.499
Yes	73 (73%)	35 (70%)	38 (76%)	
Dietary Control				
No	67 (67%)	34 (68%)	33 (66%)	0.832
Yes	33 (33%)	16 (32%)	17 (34%)	
Family History of Lipedema				
No	31 (31%)	13 (26%)	18 (36%)	0.004^1^
Yes	69 (69%)	37 (74%)	32 (64%)	
-Mother	34 (49.3%)	18 (48.8%)	16 (50%)	
-Siblings	12 (17.4%)	5 (13.5%)	7 (21.9%)	
-Grandmother	5 (7.2%)	3 (8.1%)	2 (6.3%)	
-Aunt	18 (26.1%)	11 (29.7%)	7 (21.9%)	
Relationship with Spouse				
Yes (Good)	60 (61.2%)	31 (62%)	29 (60.4%)	0.872
No (Poor)	38 (38.8%)	19 (38%)	19 (39.6%)	
Comorbidities				
No	72 (72%)	36 (72%)	36 (72%)	1
Yes	28 (28%)	14 (28%)	14 (28%)	
Medications in Use				
No	43 (43%)	18 (36%)	25 (50%)	0.157
Yes	57 (57%)	32 (64%)	25 (50%)	
Predominant Symptom				
Weight gain	51 (51%)	30 (60%)	21 (42%)	0.158^1^
Hematoma	2 (2%)	0	2 (4%)	
Aesthetic concern	37 (37%)	16 (32%)	21 (42%)	
Swelling	9 (9%)	3 (6%)	6 (12%)	
Cramps	1 (1%)	1 (2%)	0	

There was a predominance of women who engaged in regular physical activity. Regarding eating habits, 33% of the sample reported following some type of diet regularly. Most women (69%) reported a family history of signs or symptoms consistent with lipedema. Among those who responded affirmatively in both groups, the mother was the main source of information (48.8% in the placebo group and 50% in the intervention group).

Among the main complaints associated with lipedema, the sensation of heaviness in the lower limbs was the most prevalent in both groups, 60% in the placebo group and 42% in the intervention group. Aesthetic dissatisfaction was the second most frequently reported, present in 37% of the total sample. Cramps were the least frequent symptom, reported by only one participant (1%) in the placebo group.

Table 2 presents the mean QuASiL questionnaire scores and anthropometric variables at baseline. The mean QuASiL scores at baseline were similar between the two groups. Regarding anthropometric data, the mean body weight was lower in the intervention group (76.7 ± 14.5 kg) than in the control group (83.7 ± 12.9 kg) (F(1,98) = 6.608; p < 0.05). However, there were no differences in other anthropometric variables evaluated, such as BMI (F(1,98) = 3.483; p > 0.05) and measures of total and segmental body composition: % total body fat (F(1,98) = 1.070; p > 0.05), % abdominal fat (F(1,98) = 2.538; p > 0.05), % left leg fat (F(1,98) = 1.009; p > 0.05), and % right leg fat (F(1,98) = 0.984; p > 0.05). The mean fat percentages of the left (χ²(1) = 2.514; p > 0.05) and right (χ²(1) = 2.492; p > 0.05) arms were evaluated using the Kruskal-Wallis test, and no significant differences were found between groups.

**Table 2:** Baseline distribution of outcome variables. ¹ Univariate ANOVA test: values presented as mean ± SD. ² Kruskal-Wallis test: Values presented as median (minimum; maximum). QuaSiL: Quality of Life Assessment for Lipedema.

Variable	Total	Placebo	Intervention (Pycnogenol®)	p-value
QuASiL (0-150)¹	88.4 ± 23.7	88.1 ± 22.0	88.7 ± 25.5	0.89
Weight (kg)¹	80.2 ± 14.1	83.7 ± 12.9	76.7 ± 14.5	0.012
BMI (kg/m²)¹	30.3 ± 5.3	31.3 ± 4.9	29.3 ± 5.5	0.065
Body Composition (%)				
% Total Body Fat¹	40.2 ± 7.9	41.0 ± 7.3	39.4 ± 8.5	0.303
% Abdominal Fat¹	303.3 ± 91.7	317.8 ± 87.8	288.8 ± 94.3	0.114
% Left Leg Fat¹	215.9 ± 85.8	224.5 ± 82.0	207.3 ± 89.4	0.318
% Right Leg Fat¹	217.7 ± 87.4	226.4 ± 83.6	209.1 ± 91.1	0.067
% Left Arm Fat²	262.9 (69.0; 798.8)	276.5 (79.7; 771.2)	238.1 (69.0; 789.8)	0.113
% Right Arm Fat²	260.1 (66.6; 791.4)	274.5 (66.6; 763.2)	242.1 (75.2; 791.4)	0.07

**Figure 2:**
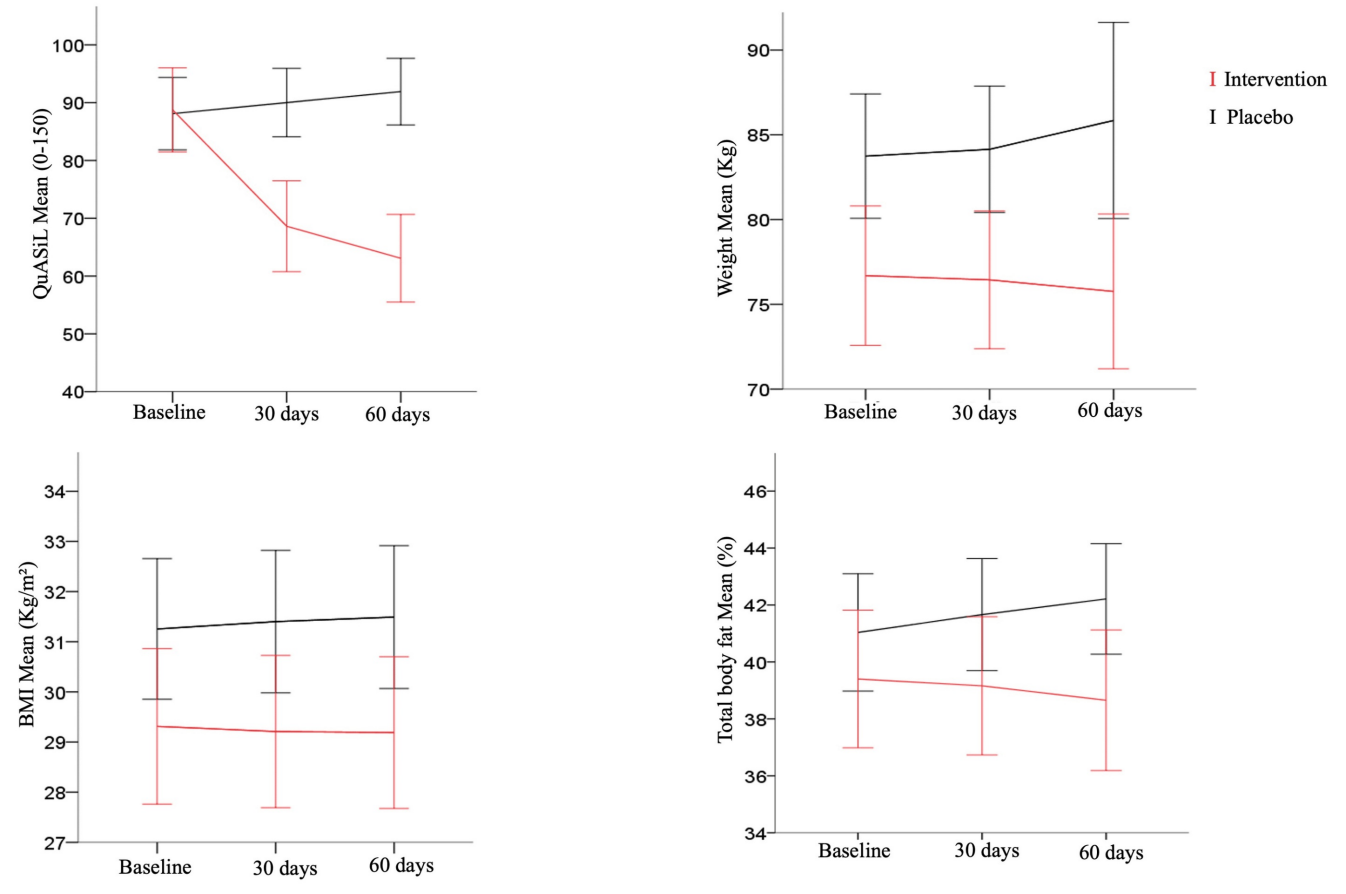
Effect of Pycnogenol® on mean QuASiL scores and anthropometric variables over time. (A) total QuASiL score; (B) body weight; (C) BMI; and (D) total body fat percentage. QuASiL: Quality of Life Assessment for Lipedema.

The effect of Pycnogenol® on anthropometric variables and the mean total QuASiL score is presented in Table 3 and Figure 2.

**Table 3:** Evolution of anthropometric parameters and QuASiL scores over time. QuASiL: Quality of Life Assessment for Lipedema.

Outcome	Placebo		Intervention (Pycnogenol®)			ANOVA for repeated measures			
	Pre	30 days	60 days	Pre	30 days	60 days	Group p-value	Time p-value	Group × Time p-value
Mean ± SD (95% CI)									
Lipedema (QuASiL 0-150)	88.1 ± 22.0 (81.8-94.4)	90.9 ± 20.6 (85.0-96.7)	92.9 ± 19.5 (87.4-98.5)	88.8 ± 25.6 (81.5-96.0)	69.5 ± 28.0 (61.5-77.4)	63.2 ± 26.9 (55.5-70.8)	0.001	<0.001	<0.001
Weight (kg)	83.7 ± 12.9 (80.1-87.4)	84.2 ± 13.1 (80.5-87.9)	84.6 ± 13.1 (80.9-88.3)	76.7 ± 14.5 (72.6-80.8)	76.5 ± 14.3 (72.4-80.5)	76.5 ± 14.2 (72.5-80.6)	0.01	0.03	0.001
BMI (kg/m²)	31.3 ± 4.9 (29.9-32.7)	31.4 ± 5.0 (29.9-32.8)	31.5 ± 5.0 (30.0-32.9)	29.3 ± 5.5 (27.8-30.9)	29.2 ± 5.4 (27.7-30.8)	29.9 ± 5.3 (27.7-30.8)	0.04	0.224	0.002
Body Fat (%)	41.0 ± 7.3 (38.9-43.1)	41.4 ± 6.9 (39.5-43.4)	42.0 ± 6.8 (40.1-43.9)	39.4 ± 8.5 (36.9-41.8)	39.4 ± 8.8 (36.8-41.9)	38.8 ± 8.9 (36.3-41.4)	0.139	0.658	0.002

A two-way repeated-measures ANOVA revealed significant differences in mean QuASiL scores between groups (F(1,49) = 13.001; p = 0.001), over time (F(1.293,63.378) = 43.881; p < 0.001), and for the time×group interaction (F(1.344,65.866) = 101.878; p < 0.001). Post hoc analysis using the Bonferroni test indicated that changes in QuASiL questionnaire scores differed significantly between groups and across time points, as shown in Figure 2A.

At baseline, there were no differences in mean QuASiL scores between the intervention and placebo groups. However, the placebo group showed a progressive increase in mean QuASiL scores at 30 and 60 days after the first evaluation, indicating a worsening of symptoms over time. In contrast, the intervention group exhibited a significant and progressive reduction in scores, with means of 69.5 ± 28 at 30 days and 63.2 ± 27 at 60 days (p < 0.001).

For body weight (Figure 2B), there was a significant effect of group (F(1,49) = 7.059; p = 0.01), time (F(2,98) = 3.518; p = 0.03), and time×group interaction (F(2,98) = 7.569; p = 0.001). Post hoc analysis showed that body weight remained stable in the intervention group throughout the study, whereas a slight increase in mean body weight was observed in the placebo group over time.

A significant time×group interaction was also found for BMI (F(2,98) = 6.873; p = 0.002). As shown in Figure 2C, similar to body weight, no variation in mean BMI was observed in the intervention group, while a slight increase occurred in the placebo group.

The time×group interaction was significant for body fat percentage (%BF) (F(1.361,66.697) = 6.533; p = 0.002). Post hoc analysis revealed that mean %BF increased in the placebo group between 30 and 60 days of the study, whereas the intervention group demonstrated a reduction in body fat percentage during the same period (Figure 2D).

**Figure 3:**
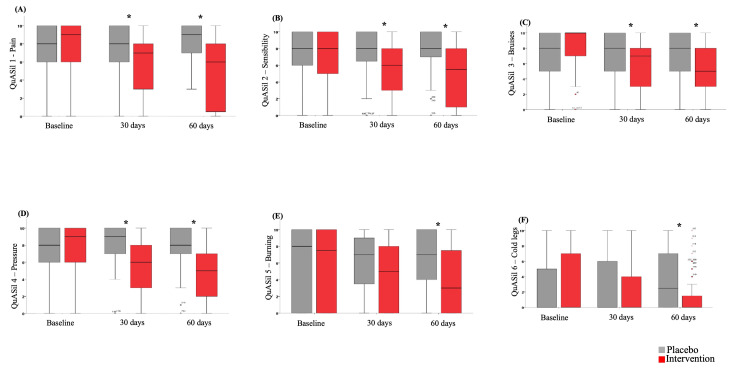
Effect of Pycnogenol® use on the median values of items one to six of the QuASiL questionnaire, analyzed separately. QuASiL: Quality of Life Assessment for Lipedema.

**Figure 4:**
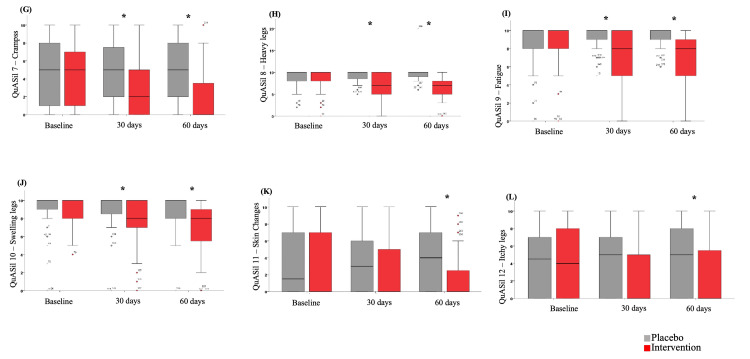
Effect of Pycnogenol® use on the median values of items 7-12 of the QuASiL questionnaire, analyzed separately. QuASiL: Quality of Life Assessment for Lipedema.

**Figure 5:**
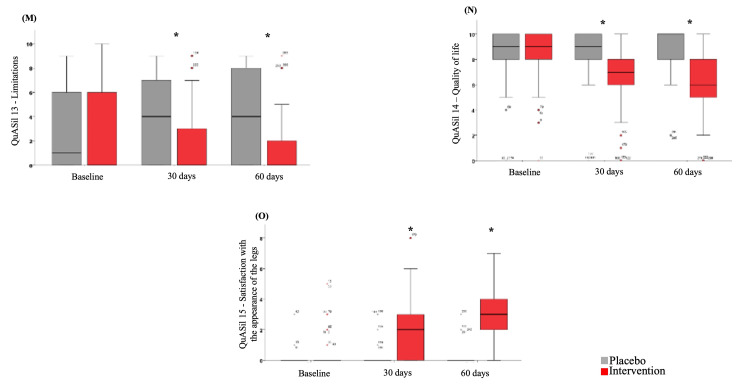
Effect of Pycnogenol® use on the median values of items 13–15 of the QuASiL questionnaire, analyzed separately. QuASiL: Quality of Life Assessment for Lipedema.

A significant effect of Pycnogenol® use was observed in each item assessed by QuASiL (Figures 3-5). When analyzing each question assessed by QuASiL separately, no differences were found in the median responses at baseline between the groups. However, after 30 days, a significant improvement was observed in the intervention group, with a reduction in the median responses related to lipedema symptoms and an increase in the median responses on questions related to quality of life and satisfaction with the appearance of the legs.

## Discussion

Lipedema has been described since the 1940s as a clinical condition related to body fat distribution in women. A preliminary search on PubMed shows that studies on the topic made little progress until 2016; since then, new research has begun to emerge. This renewed interest in the topic by the medical and scientific community is largely justified by the need for a better understanding of the disease, from its pathophysiology to available treatments [7].

For many years, this condition was underdiagnosed, in part because it was confused with diseases presenting similar signs and symptoms [1]. To address some gaps in knowledge on the topic, this clinical trial was designed to evaluate the effects of Pycnogenol®, compared with a placebo group, on quality of life and anthropometric variables in women with lipedema. After randomization, at baseline, the sample was considered homogeneous regarding age, sociodemographic variables, lifestyle habits, and lipedema-related symptoms. However, body weight showed differences between groups. There were no statistically significant differences in other anthropometric variables, such as BMI and body composition. Although body weight is a relevant parameter in disease development, both in clinical practice and research, and particularly in lipedema, measuring body weight or BMI alone is limited. Therefore, analyzing an individual's body composition and adipose tissue distribution is essential to obtain a more accurate understanding of their metabolic and functional status [7,15,16]. In this sense, it was observed that the percentage of total and segmental body fat was similar between the groups, reinforcing the homogeneity of the sample regarding body composition and ensuring that comparability between groups was not compromised.

As observed in other studies [17], participants in both groups had high percentages of body fat, especially in the upper and lower limbs. The group using Pycnogenol® experienced a significant reduction in body weight, accompanied by a slight decrease in BMI and body fat percentage over time. These findings suggest that Pycnogenol® may have additional beneficial effects on weight control and body composition, representing a positive outcome for patients with lipedema, who often face weight-related difficulties.

Regarding the variables assessed by QuASiL, after 60 days of treatment, participants in the Pycnogenol® group showed a significant reduction in lipedema-related symptoms. The mean total QuASiL questionnaire score in the intervention group decreased substantially over time compared with the placebo group, which even demonstrated a worsening of symptoms. Individual analysis of each questionnaire item allowed for a more precise assessment of the impact of Pycnogenol® on symptoms that directly affect patients’ quality of life.

Within the first 30 days of use, there was an improvement in complaints of pain and tenderness in the affected areas, bruising, tension/pressure, and a burning sensation in the legs, as well as in symptoms such as cold legs, cramps, heaviness and fatigue, edema, skin irritation, itching, and difficulty walking, with even more significant improvement observed after 60 days. As a result of the improvement in each symptom, there was a substantial enhancement in the patients’ quality of life, positively reflecting on their overall well-being and self-esteem.

It is worth noting that the latest German guideline on lipedema [18] was emphatic regarding the importance of emotional care and the impact of treatment on patients’ quality of life. In the present study, we sought to present a new therapeutic possibility that appears to have a significant positive impact on symptoms and, consequently, on patients’ quality of life.

It is also important to consider that this is a pioneering study on the use of a flavonoid as an adjuvant in the treatment of a prevalent and emotionally impactful disease. We recommend conducting more robust, multicenter studies stratified by lipedema severity to better determine which patients may benefit most from this medication.

Limitations

Potential limitations of this study include the fact that patients resided in the same region and were treated in a single clinic. There was also a difference between groups in terms of initial body weight, which could represent a source of bias. More robust, multicenter studies are needed to confirm and validate the findings presented here.

## Conclusions

The effects of Pycnogenol® on human health have been extensively investigated, with proven benefits in various clinical conditions, primarily due to its composition and main effects: antioxidant, anti-inflammatory, positive impact on blood circulation, and strengthening of the extracellular matrix. These pharmacological characteristics make the compound a promising candidate for the management of chronic diseases involving inflammation, vascular impairment, and alterations in body composition, such as lipedema.

Furthermore, studies have shown that Pycnogenol® can aid in weight loss, primarily by improving glycolipid metabolism and promoting fat burning (lipolysis), as well as helping to control risk factors associated with metabolic syndrome, such as abdominal obesity. One of the mechanisms underlying this action involves the breakdown and release of fat stored in adipocytes (fat cells) through activation of the cyclic adenosine monophosphate (cAMP)-dependent protein kinase A (PKA) pathway, which induces hormone-sensitive lipase (HSL).

This research represents, to our knowledge, the first clinical investigation to evaluate the effects of Pycnogenol® in women with a clinical diagnosis of lipedema. Although the results obtained are promising, additional multicenter clinical trials with greater methodological rigor and longer follow-up periods are necessary to confirm and expand upon the findings presented here.

In summary, the results of this study indicate that Pycnogenol® may represent an effective and safe adjuvant therapeutic alternative in the management of lipedema, contributing to the alleviation of symptoms such as pain, edema, cramps, and changes in body composition. Moreover, a positive impact on participants’ quality of life was observed, encompassing both physical and psychosocial benefits. These findings, aligned with the pharmacological profile of the standardized Pycnogenol® extract, reinforce its potential as a complementary and promising therapeutic strategy for the treatment of this clinical condition, which remains underexplored in current clinical practice.
